# Evaluation of the rapid diagnostic test SDFK40 (Pf-pLDH/pan-pLDH) for the diagnosis of malaria in a non-endemic setting

**DOI:** 10.1186/1475-2875-10-7

**Published:** 2011-01-12

**Authors:** Jessica Maltha, Philippe Gillet, Lieselotte Cnops, Emmanuel Bottieau, Marjan Van Esbroeck, Cathrien Bruggeman, Jan Jacobs

**Affiliations:** 1 Faculty of Health, Medicine and Life Sciences (FHML), Maastricht, The Netherlands; 2 Department of Clinical Sciences, Institute of Tropical Medicine (ITM), Unit of Tropical Laboratory Medicine, Antwerp, Belgium; 3 Department of Medical Microbiology, Maastricht Infection Center, School for Public Health and Primary Care: CAPHRI, Maastricht University Medical Center, The Netherlands

## Abstract

**Background:**

The present study evaluated the SD Bioline Malaria Ag 05FK40 (SDFK40), a three-band RDT detecting *Plasmodium falciparum*-specific parasite lactate dehydrogenase (Pf-pLDH) and pan *Plasmodium*-specific pLDH (pan-pLDH), in a reference setting.

**Methods:**

The SDFK40 was retrospectively and prospectively tested against a panel of stored (n = 341) and fresh (n = 181) whole blood samples obtained in international travelers suspected of malaria, representing the four *Plasmodium *species as well as *Plasmodium *negative samples, and compared to microscopy and PCR results. The prospective panel was run together with OptiMAL (Pf-pLDH/pan-pLDH) and SDFK60 (histidine-rich protein-2 (HRP-2)/pan-pLDH).

**Results:**

Overall sensitivities for *P. falciparum *tested retrospectively and prospectively were 67.9% and 78.8%, reaching 100% and 94.6% at parasite densities >1,000/μl. Sensitivity at parasite densities ≤ 100/μl was 9.1%. Overall sensitivities for *Plasmodium vivax *and *Plasmodium ovale *were 86.7% and 80.0% (retrospectively) and 92.9% and 76.9% (prospectively), reaching 94.7% for both species (retrospective panel) at parasite densities >500/μl. Sensitivity for *Plasmodium malariae *was 21.4%. Species mismatch occurred in 0.7% of samples (3/411) and was limited to non-*falciparum *species erroneously identified as *P. falciparum*. None of the *Plasmodium *negative samples in the retrospective panel reacted positive. Compared to OptiMAL and SDFK60, SDFK40 showed lower sensitivities for *P. falciparum*, but better detection of *P. ovale*. Inter-observer agreement and test reproducibility were excellent, but lot-to-lot variability was observed for pan-pLDH results in case of *P. falciparum*.

**Conclusion:**

SDFK40 performance was poor at low (≤ 100/μl) parasite densities, precluding its use as the only diagnostic tool for malaria diagnosis. SDFK40 performed excellent for *P. falciparum *samples at high (>1,000/μl) parasite densities as well as for detection of *P. vivax *and *P. ovale *at parasite densities >500/μl.

## Background

Despite increasing efforts in prevention and treatment, malaria remains a major cause of death, especially in sub-Saharan Africa [1]. Prompt and accurate diagnosis is necessary to start adequate treatment. Microscopy, the gold standard for malaria diagnosis, is labor-intensive and requires considerable expertise [2,3]. The use of malaria rapid diagnostic tests (RDTs) is recommended by the World Health Organization (WHO) when reliable microscopy is not available [4]. In non-endemic settings, where microscopic expertise is lacking due to low incidence, malaria RDTs are of value for the diagnosis of malaria and they provide information about the involvement of *Plasmodium falciparum*. In a recent external quality control session, 72.7% of 183 Belgian laboratories offering malaria diagnosis declared to use RDTs as a tool for diagnosis, and their use is recommended if performed in conjunction with microscopy [5]. RDTs are handheld cassettes detecting *Plasmodium *parasites by an antibody-antigen reaction. They are available in several formats, detecting one or more antigens. Targeted antigens are specific to *P. falciparum *(histidine-rich protein-2 (HRP-2) and *P. falciparum*-specific parasite lactate dehydrogenase (Pf-pLDH)), specific to *Plasmodium vivax *(*P. vivax*-specific pLDH (Pv-pLDH)) or common to the four *Plasmodium *species (pan-pLDH or aldolase). Most RDTs detecting *P. falciparum *target HRP-2, only few of them are directed to Pf-pLDH [6,7]. However, Pf-pLDH based RDTs have advantages over the HRP-2 based RDTs, such as the rapid clearance of pLDH after successful treatment [8], the absence of the prozone effect [9] and the fact that recently observed HRP-2 gene deletions impede detection of *P. falciparum *by HRP-2 based RDTs [10]. For routine diagnosis of malaria at the Institute of Tropical Medicine (ITM), Antwerp, Belgium, a reference laboratory in a non-endemic setting, both a HRP-2 and a Pf-pLDH RDT are performed.

SD Bioline Malaria Ag 05FK40 (Standard Diagnostics Inc., Hagal-Dong, Korea), further referred to as SDFK40, is a one-step three-band RDT targeting Pf-pLDH and pan-pLDH. The present study was performed to evaluate the test characteristics of the SDFK40 and determine its possible role in routine diagnosis.

## Methods

### Study design

The study was performed in a non-endemic reference setting and consisted of two parts: a retrospective study on stored clinical samples and a prospective study in which the SDFK40 was challenged to fresh samples and run side to side to two other RDTs, OptiMAL and SDFK60, which were used as part of routine diagnostic procedure. The reference method was microscopy corrected by PCR for malaria diagnosis and *Plasmodium *species identification; for determination of parasite density, microscopy was the reference method. The study design was in compliance with the STARD guidelines for presentation of diagnostic studies [11].

### Patients and materials

EDTA anti-coagulated blood samples were obtained in patients clinically suspected of malaria. Most patients presented at the outpatient clinic of ITM. A small part of the samples had been submitted for confirmation to ITM by other Belgian laboratories in the scope of its reference function.

For the retrospective evaluation, a selection was made out of a collection of stored whole blood samples comprising the four human *Plasmodium *species at different parasite densities. Mixed infections were not included. In addition, *Plasmodium *negative samples obtained in patients suspected of malaria were included: these samples were microscopy and PCR-negative, and showed no test lines with any of the RDTs used in routine diagnosis (OptiMAL and SDFK60). The samples had been obtained between January 2002 and April 2009 and had been stored at -70°C. Samples collected at ITM remained at room temperature (below 25°C) for a maximum of 8 hours before storing at -70°C. Samples send to ITM by other laboratories were exposed to ambient temperatures during the time of shipment which was generally less than 24 hours with a maximum of 48 hours.

The prospective part was performed between March 2009 and October 2010 and included first samples of each patient for which microscopy or one of the both routinely used RDTs (OptiMAL and SDFK60) were positive for malaria.

### Reference method

All samples were assessed by an expert microscopist for diagnosis of malaria, species identification and determination of parasite density as described previously [12]. Positive or doubtful results were repeated and confirmed by a second, blinded, expert microscopist. PCR was performed on whole blood samples for which microscopy or RDT was positive: for the retrospective part real-time PCR was adapted from Rougemont *et al *[12,13], for the prospective study a four-primer real-time PCR was performed [14]. For species identification, the result of microscopy corrected by PCR results was used as the reference method.

### Test platforms

The SDFK40 is a lateral flow antigen detection test in a cassette format, targeting Pf-pLDH and pan-pLDH. In case of absence of the control line the test is considered invalid and has to be repeated. A unique positive Pf-pLDH line represents a *P. falciparum *infection whereas a unique pan-pLDH line indicates an infection with one or more of the non-*falciparum *species. The presence of both test lines indicates either an infection with *P. falciparum *or a mixed infection with *P. falciparum *and one or more of the non-*falciparum *species.

For the evaluation of the SDFK40, RDT kits from several lot numbers were used: in the retrospective study lot numbers R081005 (n = 277) and R081006 (n = 64) were used, for the prospective evaluation lot numbers BD8002 (n = 68), BD9001 (n = 11) and R081006 (n = 101) were used. All tests had been performed at last three months before the expiry date.

The OptiMAL ^® ^pLDH (Pan, Pf) (Diamed AG Switzerland), further referred to as OptiMAL, is one of the first released three-band RDTs, targeting Pf-pLDH and pan-pLDH. The SD Bioline Ag Pf/Pan 05FK60 (Standard Diagnostics Inc.), further referred to as SDFK60, is a three-band RDT targeting HRP-2 and pan-pLDH and is in use at ITM since 2009 [12]. All RDT kits had been stored between 18°C and 24°C. Malaria diagnosis at ITM is accredited according to NBN EN ISO 15189:2007.

### Test procedures

RDTs were performed according to the manufacturer's instructions except that a transfer pipette (Finnpipette, Helsinki, Finland) was used instead of the plastic transfer devices supplied in the kit. Reading of the test results was carried out at daylight assisted by an electric bulb. For the retrospective study, readings were subsequently performed by three trained observers, of whom the first was the one performing the test. Observer 1 read the test results after 20 minutes followed by observers 2 and 3 in ten additional minutes, still within the reading time mentioned by the manufacturer's instructions (20-30 minutes). The observers were blinded to each others' readings and to the results of microscopy and PCR.

To assess line intensities, a scoring system of five categories was used as described previously [12]: none (no line visible), faint (barely visible line), weak (paler than the control line), medium (equal to the control line) or strong (stronger than the control line). The test results were based on consensus agreement, *i.e*. an identical result read by at least two out of three observers. In case of no consensus the result of the first reader was considered.

Inter-observer agreement was evaluated by determining overall agreement and kappa values for both the results in terms of positive and negative readings as well as for line intensities. To assess test reproducibility, 15 retrospective samples representing all species at variable parasite densities were tested on five consecutive occasions.

For the prospective study, the results of the routine diagnostic work-up were considered: the laboratory technician performing microscopy also performed the RDTs. RDT results were generally read before microscopy results were available. Only the results of the first observer were considered as a second and third were not always available within the given time. Results were recorded as test line intensities.

### Statistical analysis

The interpretation of test results for *P. falciparum *and the non-*falciparum *species is shown in Table 1. Samples with pure gametocytaemia were included among the *P. falciparum *species. Sensitivity and specificity were calculated with 95% confidence intervals (C.I.). Proportions were assessed for statistical significance using the Pearson Chi-square test, or in case of small sample size, the two-tailed Fisher's exact test. A *p*-value < 0.05 was considered significant.

**Table 1:** Interpretation of test results

For *P. falciparum*		
Test Line(s) visible	Species identification by microscopy corrected by PCR	
	
	*P. falciparum*^†^	*P. vivax, P. ovale, P. malariae*/no parasites detected
		
Only Pf-pLDH or both Pf-pLDH and pan-pLDH	True positive	Species mismatch**/false positive
No test line/only pan-pLDH	False negative/species mismatch*	True Negative

**For the non-*falciparum *species**		

Test Line(s) visible	Species identification by microscopy corrected by PCR	
	
	*P. vivax*, *P. ovale *or *P. malariae*	*P. falciparum*^†^/no parasites detected
		
Only pan-pLDH	True positive	Species mismatch*/false positive
No test line/only Pf-pLDH or both Pf-pLDH and pan-pLDH	False negative/species mismatch**	True Negative

* *P. falciparum *diagnosed as non-*falciparum *species

** Non-*falciparum *species diagnosed as *P. falciparum *or as a mixed infection with *P. falciparum*

^† ^Including samples with pure gametocytemia

Cramer's V for categorical variables was used to assess strength of associations between parasite densities and line intensity readings. Inter-observer agreements for line intensities and positive and negative test results were expressed by kappa values for each pair of observers and by the percentage of overall agreement between the three observers.

### Ease of use

The technicians performing the tests evaluated the SDFK40 kit's content and instructions for clarity, and problems and incidents during test performance were consequently recorded.

### Ethical review

The study was approved by the Institutional Review Board of ITM and by the Ethical Committee of Antwerp University, Belgium.

## Results

### Sample collection

The retrospective study included a selection of 341 samples obtained in 341 patients. Their median age was 34 years (range 6 months - 84 years) and eight patients (2.3%) were children under the age of five years. The male-to-female ratio was 2.25:1. The travel history was known in 86.5% (295/341) of samples: 88.1% (260/295) of patients had recently returned from Sub-Saharan Africa, 8.8% from Asia and 3.1% from Latin America. Samples consisted of all four *Plasmodium *species at different parasite densities (Tables 2, 3 and 4). For *P. falciparum *samples, the median parasite density was 1,115/μl (range 0.1 - 1,000,000/μl). For *P. vivax *this was 969/μl (range 60 - 32,000/μl), for *P. ovale *1,331/μl (range 19 - 5,930/μl) and for *P. malariae *645/μl (range 0.1 - 9,900/μl). Two out of 30 PCR-confirmed *P. vivax *samples had originally been diagnosed by microscopy as *P. ovale*, whereas 5/30 *P. ovale *samples had originally been diagnosed as *P. vivax*.

**Table 2:** Test lines visible for the SDFK40

Samples of the retrospective panel (n = 341)				
**Samples**	**Both Pf-pLDH and pan-pLDH lines**	**Only Pf-pLDH line**	**Only pan-pLDH line**	**No test line observed**
				
*P. falciparum *(n = 172)	105	6	-	61
*P. vivax *(n = 30)	-	-	26	4
*P. ovale *(n = 30)	-	-	24	6
*P. malariae *(n = 14)	2*	-	3	9
Negative^‡ ^(n = 95)	-	-	-	95

**Samples of the prospective panel (n = 180)**				

**Samples**	**Both Pf-pLDH and pan-pLDH lines**	**Only Pf-pLDH line**	**Only pan-pLDH line**	**No test line observed**
				
*P. falciparum *(n = 137)	87	21	-	29
*P. vivax *(n = 14)	-	-	13	1
*P. ovale *(n = 13)	-	1*	10	2
*P. malariae *(n = 1)	-	-	-	1
Negative^§ ^(n = 15)	2^†^	3^†^	-	10

* Species mismatch

^† ^False positive result

^‡ ^PCR and microscopy negative, routinely tested RDTs negative

^§ ^PCR and microscopy negative, routinely tested RDTs (Optimal and/or SDFK60) positive

**Table 3:** Sensitivities and specificity of the SDFK40 for the detection of *P. falciparum*, retrospective samples

**Results of microscopy corrected by PCR**	**Number**	**Identified as *P. falciparum *by SDFK40**	**% Sensitivity (95% C.I.)**	**% Specificity (95% C.I.)**
				
All *P. falciparum *samples	172	111	67.9 (60.1-75.1)	
Pure gametocytemia	13	3	23.1 (5.0-23.8)	
Asexual parasite density 0-100/μl	22	2	9.1 (1.1-29.2)	
Asexual parasite density 101-1,000/μl	55	24	43.6 (30.3-57.7)	
Asexual parasite density >1,000/μl	82	82	100.0 (95.6-100.0)	
Asexual parasite density >100/μl	137	106	77.4 (69.5-84.1)	
				
All other species and no parasites seen	169	2*		98.8 (95.8-99.9)

* Both were *P. malariae *samples

**Table 4:** Sensitivities and specificity of the SDFK40 for the detection of non-*falciparum *species, retrospective samples

**Results of microscopy corrected by PCR**	**Number**	**Identified as non-*falciparum *species by SDFK40**	**% Sensitivity (95% C.I.)**	**% Specificity (95% C.I.)**
				
All non-*falciparum *species combined	74	53	71.6 (60.0-81.5)	
Parasite density ≤ 500/μl	28	14	50.0 (30.7-69.4)	
Parasite density > 500/μl	46	39	84.8 (71.1-93.7)	
*P. vivax *samples, total	30	26	86.7 (69.3-96.2)	
Parasite density ≤ 500/μl	11	8	79.7 (39.0-94.0)	
Parasite density > 500/μl	19	18	94.7 (74.0-99.9)	
*P. ovale *samples, total	30	24	80.0 (61.4-92.3)	
Parasite density ≤ 500/μl	11	6	54.6 (23.4-83.3)	
Parasite density > 500/μl	19	18	94.7 (74.0-99.9)	
*P. malariae *samples, total*	14	3	21.4 (4.7-50.8)	
*P. falciparum *and negative samples, total	267	0		100.0 (97.9-100)

* Too few samples to calculate sensitivity according to parasite density

The panel of prospective samples (n = 181) was obtained in 180 patients, one patient had two episodes one year apart. The median age was 37 years (range 5 months - 77 years) and 3/180 (1.7%) patients were children under the age of five years. The male-to-female ratio was 1.95:1. The travel history was known in 82.2% (145/180) of samples, of which the majority (81.4%, 118/145) had been obtained in patients returned from Sub-Saharan Africa. Species and parasite densities are listed in Tables 2 and 5, median parasite density for *P. falciparum *samples was 3,993/μl (range 13 - 867,788/μl), for *P. vivax *3,367.5/μl (range 15 - 24,563/μl) and for *P. ovale *1,904/μl (range 51 - 5,310/μl). Two PCR-confirmed negative samples had originally been diagnosed by microscopy as *P. falciparum *with very low parasite densities (13/μl and 47/μl respectively). One PCR-confirmed *P. falciparum *sample had originally been concluded as negative by microscopy and was hence assigned to the category of *P. falciparum *with parasite density 0-100/μl. There was one mixed *P. falciparum/P. malariae *infection that had originally been diagnosed as *P. falciparum*. This sample was not considered for calculation of RDT accuracy.

**Table 5:** Sensitivity of the SDFK40 according to parasite density, prospective samples

**Results of microscopy corrected by PCR**	**Number**	**Correctly identified by SDFK40**	**% Sensitivity (95% C.I.)**
			
All *P. falciparum *samples	137	108	78.8 (75.3-81.1)
Pure gametocytaemia	7	3	42.9 (9.9-81.6)
Asexual parasite density 0-100/μl	11	1	9.1 (2.3-41.3)
Asexual parasite density 101-1,000/μl	26	16	61.5 (40.6-79.8)
Asexual parasite density >1,000/μl	93	88	94.6 (87.9-98.2)
Asexual parasite density >100/μl	119	104	87.4 (80.1-92.8)
			
All non-*falciparum *species combined	28	23	82.1 (72.9-82.1)
Parasite density ≤ 500/μl	6	2	33.3 (4.3-77.7)
Parasite density > 500/μl	22	21	95.5 (77.2-99.9)

### Test characteristics

No invalid test results were observed during both the retrospective and prospective studies except for a single test carried out during reproducibility testing. In two cassette blisters, discolored (pink) desiccant was found (indicating humidity saturation), they were replaced by other cassettes. Tables 2 shows the number of positive and negative test lines for all species. *P. falciparum *positive samples generated both visible Pf-pLDH and pan-pLDH test lines in 94.6% (105/111) and 80.6% (87/108) of the retrospective and prospective samples respectively (*p *= 0.0016). Twenty out of 21 prospectively tested *P. falciparum *samples that reacted only with the Pf-pLDH line had been assessed with tests of the same lot number (R081006). For this lot number a single Pf-pLDH line was observed in 29.9% (20/67) of positive tested *P. falciparum *samples, compared to 2.9% (1/34) for lot number BD8002 (*p *= 0.007), and none out of 7 for lot number BD9001 (*p *= 0.339). In the retrospective part all but one *P. falciparum *samples were tested with lot number R081005, in which a significant lower percentage (5.4%, 6/111) of positive samples generated a single Pf-pLDH line compared to lot number R081006 (*p *< 0.0002).

Tables 3 and 4 show the test characteristics for the retrospective samples. For *P. falciparum*, overall sensitivity was 67.9% ranging from 9.1% at parasite densities ≤ 100/μl to 77.4% and 100% at parasite densities above 100/μl and 1,000/μl respectively. False negative results only occurred at parasite densities below 1,000/μl, of which 39.2% (20/51) had parasite densities lower than 100/μl, and in 10/13 samples with pure gametocytaemia. Sensitivity for the non-*falciparum *species combined was 71.6%, reaching 84.8% at parasite densities >500/μl. For *P. vivax*, *P. ovale *and *P. malariae *overall sensitivities were 86.7%, 80.0% and 21.4% respectively, increasing to 94.7% at parasite densities >500/μl for both *P. vivax *and *P. ovale*. For *P. malariae*, there were not enough samples to calculate sensitivity at parasite densities >500/μl.

Table 5 shows the sensitivity of the SDFK40 when evaluated prospectively. Sensitivities were slightly higher compared to the retrospective results: overall sensitivities for *P. falciparum *and for the non-*falciparum *species combined were 78.8% and 82.1% respectively. As for the retrospective study, sensitivities were higher at increasing parasite densities. Of note, 5/93 *P. falciparum *samples with parasite densities >1,000/μl gave false negative results; the highest parasite density among these samples was 3,212/μl.

Species mismatch occurred in three samples, representing 0.7% of the pooled retrospective and prospective *Plasmodium *positive samples (n = 411): two *P. malariae *samples (parasite density of 62/μl and 9,900/μl) and one *P. ovale *sample (parasite density 113/μl) reacted not only with the pan-pLDH line, but also showed a faint Pf-pLDH line.

Compared to the OptiMAL and the SDFK60 (Table 6), the SDFK40 had a significant lower sensitivity for detection of *P. falciparum *(*p *= 0.004 and *p *< 0.0001 respectively). For the non-*falciparum *species combined, overall sensitivity of the SDFK40 tended to be higher compared to the OptiMAL and the SDFK60, though not statistically significant. Of note, the SDFK40 performed significantly better in the detection of *P. ovale *compared to the OptiMAL (*p *= 0.005) and tended to perform better than the SDFK60 (*p *= 0.226). The *P. vivax *and *P. ovale *samples that were not detected by the SDFK40 in the prospective part (n = 3) had low parasite densities (51/μl, 176/μl and 304/μl) and were tested with the same lot number (R081006), as well as the single *P. malariae *sample (parasite density 1,920/μl) that tested negative.

**Table 6:** Diagnostic accuracy of the different RDTs for each species, prospective samples

**Species**	**RDT brand**	**Number of samples tested**	**Correctly identified by RDT (number)**	**% Sensitivity (95% CI)**
				
*P. falciparum*	SDFK40	137	108	78.8 (75.3-81.1)
	OptiMAL	137	125	91.2 (87.7-93.9)
	SDFK60	137	136	99.3 (96.0-99.9)
				
Non-*falciparum*	SDFK40	28	23	82.1 (72.9-82.1)
	OptiMAL	28	17	60.7 (40.6-78.5)
	SDFK60	28	20	71.4 (51.3-86.8)
				
*P. vivax*	SDFK40	14	13	92.9 (66.1 - 99.8)
	OptiMAL	14	14	100 (76.8-100)
	SDFK60	14	13	92.9 (66.1 - 99.8)
				
*P. ovale*	SDFK40	13	10^‡^	76.9 (46.2-95.0)
	OptiMAL	13	2	15.4 (1.9-45.6)
	SDFK60	13	6	46.2 (19.2-74.9)
				
P. malariae*	SDFK40	1	0	
	OptiMAL	1	1	
	SDFK60	1	1	
				
*Plasmodium *negative^†^	SDFK40	15	10	
	OptiMAL	15	3	
	SDFK60	15	4	

* Too few samples to calculate sensitivity

^† ^Included samples were negative by microscopy and PCR, but positive by the OptiMAL and/or the SDFK60

^‡ ^Incorrect diagnosis consisted of two samples diagnosed as *Plasmodium *negative and one sample diagnosed as a single *P. falciparum *or a mixed infection of *P. falciparum *with one or more of the non-*falciparum *species.

The mixed *P. falciparum/P. malariae *infection was found in a 11-year old child returning from Cameroon and had a parasite density of 23/μl. The sample was scored as negative by the SDFK40, as a mixed infection by OptiMAL, and as a single *P. falciparum *infection by the SDFK60.

In the retrospective study none of the 95 *Plasmodium *negative samples generated positive test results (Table 2). In the prospective study, there were five false positive results among the 15 samples that were microscopy and PCR negative but positive for one or both routinely performed RDTs (Tables 2). Two of these samples tested positive for *Schistosoma *antibodies, one patient had been partially treated for malaria and the remaining two samples were from a patient (tested in 2009 and 2010 after two different journeys) in whom the RDT results were interpreted as false positive without a plausible explanation.

### Presence and intensity of test lines

For both test lines there was a correlation between parasite density and line intensity readings, though higher for the retrospective samples (Pf-pLDH: V = 0.6622 and V = 0,4814, both p < 0.0001; pan-pLDH: V = 0.6312 and V = 0,4263, both p < 0.0001). Among the pooled retrospective and prospective *P. falciparum *samples, 17.8% (39/219) of the positive Pf-pLDH test lines were read as faint. For the pooled non-*falciparum *samples, faint test lines were observed in 22.8% (18/79) of positive pan-pLDH lines. For both Pf-pLDH and pan-pLDH test lines, medium and strong intensities occurred exclusively at parasite densities above 1,000/μl, except for one sample with pure gametocytaemia.

### Reproducibility and inter-observer agreement

For both Pf-pLDH and pan-pLDH test lines overall agreement and kappa values between pairs of observers were excellent for both positive and negative readings (≥ 94.7%, kappa values ≥ 0.92) and line intensity readings (≥ 86.5%, kappa values ≥ 0.84). Test results were reproducible and all discordances in line intensity occurred within one category of difference.

### Ease of use

The SDFK40 was evaluated as easy to use, with an excellent clearance of the background and clearly visible test lines. In most tests the background became blurred after 60 minutes and test lines were no longer visible. The test instructions in the package insert were clear and accompanied by well-designed pictures. The part presenting invalid test results (absence of control line) failed to demonstrate pictures of a pan line without control line as well as both test lines without control line.

## Discussion

In this study, the performance of the malaria RDT SDFK40 was evaluated on a panel of stored (n = 341) and fresh (n = 180) whole blood samples, obtained in returned international travelers suspected of malaria. Sensitivity was poor for *P. falciparum *samples at low parasite densities, but excellent for detection of *P. falciparum *at high parasite densities, as well as for *P. vivax *and *P. ovale*.

The present study has its limitations. Although evaluation of a RDT in a standardized laboratory is a logical step preceding studies in field settings [15], it is clear that conditions in a reference setting such as the present one are more favorable compared to those in field settings (*e.g*. temperature control, training and expertise of staff). Another limitation was that, for the retrospective samples, it was impossible to explore the cause of discordant results. Furthermore stringent criteria were used. For instance, species mismatch was considered as a false negative result, although the diagnosis of malaria had been confirmed by the SDFK40: considering the non-*falciparum *species that reacted not only with the pan-pLDH line but also with the Pf-pLDH line as true positives would have increased the sensitivity for *P. ovale *to 84.6% (prospective study) and for *P. malariae *to 35.7% (retrospective study). In addition, samples with pure gametocytaemia were included among the *P. falciparum *samples [16]: assigning them to the *Plasmodium *negative samples would have increased overall sensitivity to 71.9% and 80.8% for the retro- and prospective samples respectively, at the expense however of a lower specificity (97.3%) in the retrospective panel. Another limitation was that, in the prospective study only the results of a single observer were considered, as a second and a third observer could not always perform reading within the given time. However, this probably has not influenced test results as the retrospective study revealed high inter-observer agreements. Finally, in the prospective study there was no *Plasmodium *negative control group (microscopy and RDT negative) included, precluding calculation of diagnostic specificity and predictive values.

The SDFK40 has been evaluated in two other studies: in the first round of RDT evaluation by the WHO, detection of *P. falciparum *and *P. vivax *was assessed using diluted samples at fixed parasite densities. Detection rates for *P. falciparum *and *P. vivax *were 29.1% and 50.0% at low (200/μl) parasite densities and 96.2% and 100.0% at high (2,000/μl and 5,000/μl) parasite densities respectively [6]. In a field setting in Madagascar, overall sensitivities were 89.4% for the detection of *P. falciparum *and 73.3% for the non-*falciparum *species [17]. Conform the present study, there was a higher detection rate for *P. vivax *(8/9 samples) compared to *P. malariae *(3/6 samples).

Data assessing the test characteristics of pLDH RDTs for non-*falciparum *infections are limited [18], but the low sensitivity for diagnosis of *P. malariae *and *P. ovale *is a well-known phenomenon among RDTs [16,19-21]. Unlike the previous studies, the present one included samples of *P. ovale*. The high sensitivity of SDFK40 for detection of *P. ovale *is remarkable and exceeds that of other RDTs evaluated in non-endemic settings [16,20-22] as well as that of the OptiMAL and SDFK60 run simultaneously. In the present setting at ITM, a comparable sensitivity (76.3%) for *P. ovale *has only been achieved for the SDFK60 [12]. Although species mismatches were rare and did not involve misidentification of *P. falciparum *as non-*falciparum *species, the cross-reaction of two *P. malariae *samples and one *P. ovale *sample with the Pf-pLDH should be taken into account and further studied.

The side-to-side comparison with the OptiMAL and the SDFK60 revealed significantly lower overall sensitivities for the detection of *P. falciparum *by the SDFK40. HRP-2 is known to be detected at lower parasite rates compared to pLDH [2] and this might explain for the difference in sensitivity between the SDFK40 and the SDFK60, as the latter tested positive for all *P. falciparum *samples at parasite density 0-100/μl, compared to only 1/11 for the SDFK40. Compared to the OptiMAL, the SDFK40 showed a lower sensitivity for *P. falciparum *but a higher sensitivity for the non-*falciparum *species, especially *P. ovale*. These differences may be explained by differences in affinity of antibodies [2] or by reaction conditions created by the buffer composition favoring the pan-pLDH antigen-antibody interaction over that of Pf-pLDH in the case of the SDFK40.

From the present study, it is difficult to estimate the incidence of false positive RDT results in the SDFK40: in the retrospective part, none of the *Plasmodium *negative samples reacted positive. These samples had also been tested negative by the routinely performed RDTs. In contrast, the prospective panel included microscopy negative samples that had given positive results with one or both of the routinely used RDTs (the SDFK60 and the OptiMAL) for diagnostic work-up, which may explain for discordance in false positive results between the prospective and retrospective panel. Among these samples, false positive reactions occurred less frequently in the SDFK40 compared to the OptiMAL and the SDFK60. For the SDFK60, persistence of HRP-2 may explain for part of the false positive results. The pLDH enzyme - expressed by viable parasites - declines rapidly after therapy but is also expressed by gametocytes [2,23]. Prospective monitoring of diagnostic characteristics on large series will be needed to get reliable estimates of the number of false positive SDFK40 results.

Test line intensities of the SDFK40 correlated to parasite densities, but there was much overlap, precluding reliance on line intensities as an indicator of parasite density. The test line intensity of about one out of five positive test lines was scored as faint. In the present study, observers were trained to distinguish faint test lines and to interpret them as positive; in field settings however, disregarding faint test lines as negative is a well known error that might interfere with correct reading, especially at unfavorable light conditions [24-27].

Comparison of the retro- and prospective panels revealed some differences. In the prospective panel, the overall sensitivity of the SDFK40 for the diagnosis of *P. falciparum *was higher as compared to the retrospective panel. This may be attributed to differences in parasite density between both panels: 52.3% (90/172) of *P. falciparum *samples in the retrospective panel had a parasite density ≤ 1,000/μl compared to only 32.1% (44/137) in the prospective panel (Tables 3 and 5), whereas sensitivities at low (≤ 100/μl) and high (>1,000/μl) parasite densities were similar for both panels. Although the difference in parasite density appears to be the most plausible explanation, an influence of sample storage cannot be excluded. In a previous study [20], we showed evidence for decline of parasite pLDH in the case of *P. ovale*, but this effect was noted for samples stored for extended periods (more than 8 years) which were not included in the present study. Another difference was the presence of a single test line versus both test lines in the case of *P. falciparum *samples: in the retrospective panel, the majority of *P. falciparum *samples showed both Pf-pLDH and pan-pLDH lines, while this proportion was significantly lower in the prospective panel. The presence of a single Pf-pLDH line occurred almost exclusively among samples tested with a single lot (R081006). Lot number R081006 had been used in the retrospective study for only one *P. falciparum *sample, making definite comparisons impossible; nevertheless a lot-related phenomenon is highly suggestive. Lot-to-lot variability in RDTs is a well-known phenomenon and is an issue in RDT performance monitoring and quality control [6,7,28,29]: according to WHO guidelines, each new lot number of a RDT used in diagnosis should be evaluated by a reference laboratory before release [30].

Compared to HRP-2 RDTs, pLDH-based RDTs are generally reported to have lower sensitivities for the detection of *P. falciparum*, especially at parasite densities ≤ 100/μl [2,31,32]. In addition, pLDH RDTs have been reported to be less resistant to heat degradation in comparison to HRP-2 RDTs [33], although the SDFK40 package insert claims heat stability up to 40°C and the heat stability testing of the WHO/FIND study confirmed resistance to temperatures up to 45°C [6]. On the other hand, pLDH RDTs have advantages over the HRP-2 based RDTs: as mentioned above, pLDH, unlike HRP-2, does not persist long after treated or past *P. falciparum *infections, and unlike HRP-2 based RDTs, they are not susceptible to the prozone phenomenon, *i.e*. the occurrence of test negative or low-intensity test lines at elevated parasite densities [9]. In addition, HRP-2 gene deletions and polymorphisms have been described, possibly limiting the use of HRP-2 based antigen tests in the affected areas [10,34,35]. For pLDH such genetic variability has not been described [36].

For the non-endemic setting, the low sensitivity of the SDFK40 for the detection of *P. falciparum *at parasite densities below 1,000/μl is of concern and precludes its use in routine diagnosis, as non-immune travelers may be symptomatic at such low parasite densities [2]. However, the particular advantage of the SDFK40 in this setting is its high sensitivity for both *P. vivax *and *P. ovale*, but it should be used in conjunction with another brand known for reliable detection of *P. falciparum*.

## Conclusion

Challenged to a panel of clinical samples in a reference setting, the SDFK40 showed a low sensitivity for detection of *P. falciparum *samples at parasite densities ≤ 100/μl, precluding its use as the only diagnostic tool for malaria diagnosis. However, SDFK40 performed excellent for *P. falciparum *samples at high (>1,000/μl) parasite densities as well as for detection of *P. vivax *and *P. ovale *at parasite densities >500/μl.

## List of abbreviations

CI: Confidence interval; EDTA: Ethylene diamine tetra-acetic acid; HRP-2: Histidine-rich protein 2; ITM: Institute of Tropical Medicine; *P*.: *Plasmodium*; pan-pLDH: pan *Plasmodium*-specific parasite lactate dehydrogenase; PCR: Polymerase chain reaction; Pf-pLDH: *Plasmodium falciparum*-specific parasite lactate dehydrogenase; pLDH: *Plasmodium*-specific parasite lactate dehydrogenase; Pv-pLDH: *Plasmodium vivax*-specific parasite lactate dehydrogenase; RDT(s): Rapid diagnostic test(s); STARD: Standards for reporting of diagnostic accuracy; WHO: World Health Organization.

## Competing interests

The authors declare that they have no competing interests.

## Authors' contributions

JM, PG and JJ designed the study protocol. MVE and EB organized prospective sample collection. JM and PG carried out the test evaluations, LC performed PCR analysis. JM, PG and JJ analysed and interpreted the results. JM performed statistical analysis and drafted the manuscript. JJ and CB contributed to the drafting of the manuscript. All authors read and approved the final manuscript.

## References

[B1] WHO World Malaria Report 2009 2009 World Health Organization

[B2] Murray CK Gasser RA Jr Magill AJ Miller RS Update on rapid diagnostic testing for malaria Clin Microbiol Rev 2008 21 97 110 10.1128/CMR.00035-07 18202438 PMC2223842

[B3] Moody A Rapid diagnostic tests for malaria parasites Clin Microbiol Rev 2002 15 66 78 10.1128/CMR.15.1.66-78.2002 11781267 PMC118060

[B4] WHO Guidelines for the treatment of malaria 2010 second World Health Organization

[B5] Gillet P Mukadi P Vernelen K Van Esbroeck M Muyembe JJ Bruggeman C Jacobs J External quality assessment on the use of malaria rapid diagnostic tests in a non-endemic setting Malar J 2010 9 359 10.1186/1475-2875-9-359 21144034 PMC3019163

[B6] WHO Malaria Rapid Diagnostic Test Performance Results of WHO product testing of malaria RDTs, Round 1, 2008 2009 World Health Organization

[B7] WHO Malaria Rapid Diagnostic Test Performance Results of WHO product testing of malaria RDTs: Round 2, 2009 2010 World Health Organization

[B8] Iqbal J Siddique A Jameel M Hira PR Persistent histidine-rich protein 2, parasite lactate dehydrogenase, and panmalarial antigen reactivity after clearance of Plasmodium falciparum monoinfection J Clin Microbiol 2004 42 4237 4241 10.1128/JCM.42.9.4237-4241.2004 15365017 PMC516301

[B9] Gillet P Mori M Van Esbroeck M Vanden Ende J Jacobs J Assessment of the prozone effect in malaria rapid diagnostic tests Malar J 2009 8 271 10.1186/1475-2875-8-271 19948018 PMC2789093

[B10] Gamboa D Ho MF Bendezu J Torres K Chiodini PL Barnwell JW Incardona S Perkins M Bell D McCarthy J A large proportion of *P. falciparum *isolates in the Amazon region of Peru lack pfhrp2 and pfhrp3: implications for malaria rapid diagnostic tests PLoS One 2010 5 e8091 10.1371/journal.pone.0008091 20111602 PMC2810332

[B11] Bossuyt PM Reitsma JB Bruns DE Gatsonis CA Glasziou PP Irwig LM Lijmer JG Moher D Rennie D de Vet HC Toward complete and accurate reporting of studies of diagnostic accuracy. The STARD initiative Am J Clin Pathol 2003 119 18 22 10.1309/8EXCCM6YR1THUBAF 12520693

[B12] Van der Palen M Gillet P Bottieau E Cnops L Van Esbroeck M Jacobs J Test characteristics of two rapid antigen detection tests (SD FK50 and SD FK60) for the diagnosis of malaria in returned travellers Malar J 2009 8 90 10.1186/1475-2875-8-90 19416497 PMC2688521

[B13] Rougemont M Van Saanen M Sahli R Hinrikson HP Bille J Jaton K Detection of four Plasmodium species in blood from humans by 18S rRNA gene subunit-based and species-specific real-time PCR assays J Clin Microbiol 2004 42 5636 5643 10.1128/JCM.42.12.5636-5643.2004 15583293 PMC535226

[B14] Cnops L Jacobs J Van Esbroeck M Validation of a four-primer real-time PCR as a diagnostic tool for single and mixed Plasmodium infections Clin Microbiol Infect 2010 20718798 10.1111/j.1469-0691.2010.03344.x

[B15] Bell D Peeling RW Pacific/TDR WH-ROftW Evaluation of rapid diagnostic tests: malaria Nat Rev Microbiol 2006 4 9 Suppl S34 38 10.1038/nrmicro1524 17034070

[B16] Marx A Pewsner D Egger M Nuesch R Bucher HC Genton B Hatz C Juni P Meta-analysis: accuracy of rapid tests for malaria in travelers returning from endemic areas Ann Intern Med 2005 142 836 846 15897534 10.7326/0003-4819-142-10-200505170-00009

[B17] Ratsimbasoa A Randriamanantena A Raherinjafy R Rasoarilalao N Menard D Which malaria rapid test for Madagascar? Field and laboratory evaluation of three tests and expert microscopy of samples from suspected malaria patients in Madagascar Am J Trop Med Hyg 2007 76 481 485 17360871

[B18] Murray CK Bennett JW Rapid Diagnosis of Malaria Interdiscip Perspect Infect Dis 2009 2009 415953 19547702 10.1155/2009/415953 PMC2696022

[B19] Grobusch MP Hanscheid T Zoller T Jelinek T Burchard GD Rapid immunochromatographic malarial antigen detection unreliable for detecting *Plasmodium malariae *and *Plasmodium ovale* Eur J Clin Microbiol Infect Dis 2002 21 818 820 10.1007/s10096-002-0831-0 12461593

[B20] Maltha J Gillet P Bottieau E Cnops L van Esbroeck M Jacobs J Evaluation of a rapid diagnostic test (CareStart Malaria HRP-2/pLDH (Pf/pan) Combo Test) for the diagnosis of malaria in a reference setting Malar J 2010 9 171 10.1186/1475-2875-9-171 20565816 PMC2906498

[B21] van Dijk DP Gillet P Vlieghe E Cnops L Van Esbroeck M Jacobs J Evaluation of the Immunoquick+4 malaria rapid diagnostic test in a non-endemic setting Eur J Clin Microbiol Infect Dis 2010 29 577 583 10.1007/s10096-010-0898-y 20232100

[B22] van Dijk DP Gillet P Vlieghe E Cnops L van Esbroeck M Jacobs J Evaluation of the Palutop+4 malaria rapid diagnostic test in a non-endemic setting Malar J 2009 8 293 10.1186/1475-2875-8-293 20003378 PMC2797810

[B23] Mueller I Betuela I Ginny M Reeder JC Genton B The sensitivity of the OptiMAL rapid diagnostic test to the presence of *Plasmodium falciparum *gametocytes compromises its ability to monitor treatment outcomes in an area of Papua New Guinea in which malaria is endemic J Clin Microbiol 2007 45 627 630 10.1128/JCM.00816-06 17135432 PMC1829017

[B24] Harvey SA Jennings L Chinyama M Masaninga F Mulholland K Bell DR Improving community health worker use of malaria rapid diagnostic tests in Zambia: package instructions, job aid and job aid-plus-training Malar J 2008 7 160 10.1186/1475-2875-7-160 18718028 PMC2547110

[B25] Mayxay M Newton PN Yeung S Pongvongsa T Phompida S Phetsouvanh R White NJ An assessment of the use of malaria rapid tests by village health volunteers in rural Laos Trop Med Int Health 2004 9 325 329 10.1111/j.1365-3156.2004.01199.x 14996360

[B26] Rennie W Phetsouvanh R Lupisan S Vanisaveth V Hongvanthong B Phompida S Alday P Fulache M Lumagui R Jorgensen P Bell D Harvey S Minimising human error in malaria rapid diagnosis: clarity of written instructions and health worker performance Trans R Soc Trop Med Hyg 2007 101 9 18 10.1016/j.trstmh.2006.03.011 17049572

[B27] Wiese L Bruun B Baek L Friis-Moller A Gahrn-Hansen B Hansen J Heltberg O Hojbjerg T Hornstrup MK Kvinesdal B Gomme G Kurtzhals JA Bedside diagnosis of imported malaria using the Binax Now malaria antigen detection test Scand J Infect Dis 2006 38 1063 1068 10.1080/00365540600818011 17148078

[B28] Mason DP Kawamoto F Lin K Laoboonchai A Wongsrichanalai C A comparison of two rapid field immunochromatographic tests to expert microscopy in the diagnosis of malaria Acta Trop 2002 82 51 59 10.1016/S0001-706X(02)00031-1 11904103

[B29] Coleman RE Maneechai N Rachapaew N Kumpitak C Soyseng V Miller RS Thimasarn K Sattabongkot J Field evaluation of the ICT Malaria Pf/Pv immunochromatographic test for the detection of asymptomatic malaria in a *Plasmodium falciparum/vivax *endemic area in Thailand Am J Trop Med Hyg 2002 66 379 383 12164291 10.4269/ajtmh.2002.66.379

[B30] Malaria Rapid Diagnostic Tests: Quality Assurance http://www.wpro.who.int/sites/rdt/using_rdts/qa/lot_testing.htm

[B31] Fogg C Twesigye R Batwala V Piola P Nabasumba C Kiguli J Mutebi F Hook C Guillerm M Moody A Guthmann JP Assessment of three new parasite lactate dehydrogenase (pan-pLDH) tests for diagnosis of uncomplicated malaria Trans R Soc Trop Med Hyg 2008 102 25 31 10.1016/j.trstmh.2007.09.014 18031779

[B32] Craig MH Bredenkamp BL Williams CH Rossouw EJ Kelly VJ Kleinschmidt I Martineau A Henry GF Field and laboratory comparative evaluation of ten rapid malaria diagnostic tests Trans R Soc Trop Med Hyg 2002 96 258 265 10.1016/S0035-9203(02)90092-1 12174773

[B33] Chiodini PL Bowers K Jorgensen P Barnwell JW Grady KK Luchavez J Moody AH Cenizal A Bell D The heat stability of Plasmodium lactate dehydrogenase-based and histidine-rich protein 2-based malaria rapid diagnostic tests Trans R Soc Trop Med Hyg 2007 101 331 337 10.1016/j.trstmh.2006.09.007 17212967

[B34] Baker J McCarthy J Gatton M Kyle DE Belizario V Luchavez J Bell D Cheng Q Genetic diversity of *Plasmodium falciparum *histidine-rich protein 2 (PfHRP2) and its effect on the performance of PfHRP2-based rapid diagnostic tests J Infect Dis 2005 192 870 877 10.1086/432010 16088837

[B35] Mariette N Barnadas C Bouchier C Tichit M Menard D Country-wide assessment of the genetic polymorphism in *Plasmodium falciparum *and *Plasmodium vivax *antigens detected with rapid diagnostic tests for malaria Malar J 2008 7 219 10.1186/1475-2875-7-219 18957099 PMC2582241

[B36] Talman AM Duval L Legrand E Hubert V Yen S Bell D Le Bras J Ariey F Houze S Evaluation of the intra- and inter-specific genetic variability of Plasmodium lactate dehydrogenase Malar J 2007 6 140 10.1186/1475-2875-6-140 17961215 PMC2194689

